# Approach to Design of Potent RNA Interference-Based Preparations Against Hepatocellular Carcinoma-Related Genes

**DOI:** 10.3390/ijms27020603

**Published:** 2026-01-07

**Authors:** Petr V. Chernov, Vladimir N. Ivanov, Nikolai A. Dmitriev, Artem E. Gusev, Valeriia I. Kovchina, Ivan S. Gongadze, Alexander V. Kholstov, Maiia V. Popova, Dmitry A. Kudlay, Daria S. Kryuchko, Ilya A. Kofiadi, Musa R. Khaitov

**Affiliations:** 1 NRC Institute of Immunology FMBA of Russia, 115522 Moscow, Russia; 496ram496@gmail.com (P.V.C.); kvi-91@mail.ru (V.I.K.); ivan.gongadze@gmail.com (I.S.G.);; 2 A.P. Nelyubin Institute of Pharmacy, I.M. Sechenov 1st Moscow State Medical University of the MOH of Russia (Sechenov University), 119992 Moscow, Russia; 3 Faculty of Bioengineering and Bioinformatics, M.V. Lomonosov Moscow State University, 119991 Moscow, Russia; 4 Federal Medical-Biological Agency, 123182 Moscow, Russia; 5 The Institute of Neonatology and Pediatrics, National Medical Research Center for Obstetrics, Gynecology and Perinatology Named After Academician V.I. Kulakov of Ministry of Healthcare of Russian Federation, 117198 Moscow, Russia; 6 Neonatology Department, National Medical Research Center for Obstetrics, Gynecology and Perinatology Named After Academician V.I. Kulakov of Ministry of Healthcare of Russian Federation, 117997 Moscow, Russia; 7 Medical Biological Faculty, Pirogov Russian National Research Medical University, 117513 Moscow, Russia

**Keywords:** siRNA, RNAi, hepatocellular carcinoma, HCC, ITGB1, CD47, in silico drug design

## Abstract

Every year, the scientific community continues to drive advances in healthcare, opening up new perspectives in the treatment and management of various diseases. Despite vast strides being made in the quality of life and longevity, we still face an equally significant growth in the burden of oncological pathologies. Although current trends lean towards preventive and personalized medicine, numerous hurdles remain to be cleared to develop robust strategies in the field of oncology. Among all types of tumors, one of the prominent positions is occupied by hepatocellular carcinoma (HCC), which is one of the most widespread primary cancers with a high mortality rate. Conventional approaches to HCC therapy, such as surgery or chemotherapy, rarely provide steady performance due to the highly polymorphous nature of the cancerous process. In this study, we suggest an alternative methodological framework for designing potent siRNAs targeting genes implicated in hepatocellular carcinoma, implementing RNA interference mediated by synthetic small interfering RNAs (siRNAs) against mRNAs of *ITGB1* and *CD47* genes. Products of these genes are renowned drivers of tumor progression. We have developed a software algorithm for the design of unmodified and modified siRNAs, carried out solid-phase synthesis of the most promising molecules, and proved their capability to perform a more than 50-fold suppression of expression of the target genes in vitro.

## 1. Introduction

Hepatocellular carcinoma (HCC) is an oncological pathology characterized by the malignization of hepatocytes, the formation of tumors, and metastases. HCC is one of the most prevalent primary hepatic cancers, ranking fourth in cancer-related mortality causes worldwide [1]. Akin to the majority of oncological processes, HCC may be characterized according to the Hallmarks of cancer [2]. The pathogenesis is tightly linked to the dysregulation of cellular metabolism and genetic instability which provide neoplastic cells with the means to become self-sustained in proliferative signaling, to evade growth suppressors, avoid immune destruction, induce neoangiogenesis, and resist cell death, promoting their replicative immortality and persistence in the organism, their invasion, and the formation of metastases.

Existing approaches to the therapy of HCC include surgical and cytoreductive manipulations, radiotherapy, and chemotherapy, including immunotherapy and their combinations [3,4]. Among the most promising approaches to date are the inhibitors of tyrosine kinases (lenvotinib, sorafinib) and immunotherapeutic solutions—monoclonal antibodies acting along PD-1/PD-1L (pembrolizumab, camrelizumab, atezolizumab) and CTLA4 axes (tremelimumab) [5]. However, due to their high adaptive capabilities, tumor cells rapidly modify their expression repertoires in order to avoid the action of these drugs, achieving resistance to the therapy. Therefore, new approaches are in high demand, enabling targeted suppression of the genes related to the pathogenesis of the tumor.

RNA interference (RNAi) is a physiological mechanism of regulation of gene expression acting at the level of translation. Phylogenetically, this process arose in order to protect the host’s genetic material from harmful alterations and to allow for quick adaptive changes to the environment [6]. In healthy cells, this process allows for quick differentiation and polarization in order to maintain both cellular and organismic homeostasis. However, in neoplastic lesions, malignant cells frequently implement it to further avoid systemic regulation and become self-sustained [7]. Physiological actors of RNAi are microRNAs, short non-coding RNAs that are readily bound to Argonaute family proteins, enabling the formation of an RNA-induced silencing complex (RISC). During the process of interference, targeted mRNA is degraded or ribosome dissociation from it is induced, resulting in the termination of translation and a decrease in the amount of protein product.

Nevertheless, scientific advances paved the path to harness the RNAi process, introducing small interfering RNAs (siRNAs), synthetic replicas of microRNAs. siRNAs, however, possess several crucial distinctions from their endogenous counterparts. MicroRNAs possess quite a peculiar structure, being functionally delineated into three significant regions—the “seed” region exhibits complete complementarity to the target region of the mRNA transcript, followed by the “mismatch” region and the “supplementary” region. This structure is tailored to induce RNAi on 3′-UTR segments of mRNAs which are highly homologous to one another [8], making the physiological process efficient, yet imprecise. On the other hand, antisense strands of siRNAs exhibit complete complementarity to the targeted sequence of the mRNA transcript, enabling gene-specific silencing within the coding sequence, allowing for the targeted silencing of the expression of the specific genes.

To date, the field of drugs employing RNAi mechanisms is seeing an explosive growth. Numerous preparations are being developed on the basis of siRNAs as well as antisense oligonucleotides (ASOs) [9]. In addition, new avenues are being explored researching the inactivation of pathogenic microRNAs by antagoMIRs [10]. Already, several drugs are approved by the FDA, and some are being included in the list of essential medicines, becoming a mainstay in the therapy of orphan diseases. However, one common trend may be outlined—RNAi drugs follow the paths paved by rigorous research of monoclonal antibodies and small molecular drugs.

Contemporary successful approaches to HCC therapy are likewise based on the action of monoclonal antibodies. Specifically, the binding of surface moieties like ITGB1 and CD47 show great promise, exhibiting not only tumoristatic but also tumoricidal effects [11]. Researchers demonstrate that the binding of these markers ex vivo suppresses the invasion of tumor cells and the development of metastases, as well as activating cytotoxic immune response against them [12]. Studies link the observed effects to the significant role of the suppressed structures in tumor cell viability. ITGB1 is an integrin subunit which takes a crucial part in the formation of an integrin receptor, an extracellular adhesion molecule (ECAM) conferring adhesion to the intercellular matrix. Fixation of the cell in the matrix is amongst the most significant survival and proliferation stimuli to the cells. The cells unable to anchor within the matrix undergo rapid apoptosis [13,14].

An analogous approach is shown for CD47, which is highly overexpressed in neoplastic cells. This molecule, also known as integrin-associated protein (IAP), is a significant surface marker expressed by the majority of cells. CD47 acts as a ligand presented to a signal regulatory protein α (SIRPα or CD172a) molecule on the surface of macrophages. The successful presentation of CD47 confers a “do-not-eat-me” signal to the macrophage, preventing host cell lysis [15].

While ITGB1 and CD47 may not show strong differential expression between tumor and adjacent tissues, their functional role in tumor invasion, immune evasion, and metastatic competence has been repeatedly demonstrated, supporting them as functional rather than diagnostic targets. Most importantly, it appears that healthy cells exhibit redundant expression repertoires for the functions of the described molecules, which means that their inhibition on healthy cells would not significantly impact them, whereas cancerous cells appear to decrease the amount of such molecules on their surface in favor of a higher metabolic capacity, suggesting that the loss of these moieties would be fatal for them [16,17].

Given the information which lies in the foundation of the hypothesis of this research, we suggest the use of RNAi against the mRNAs of *ITGB1* and *CD47* genes in order to hinder or even abort the progression of HCC without significantly impacting the healthy cells.

**Figure 1:**
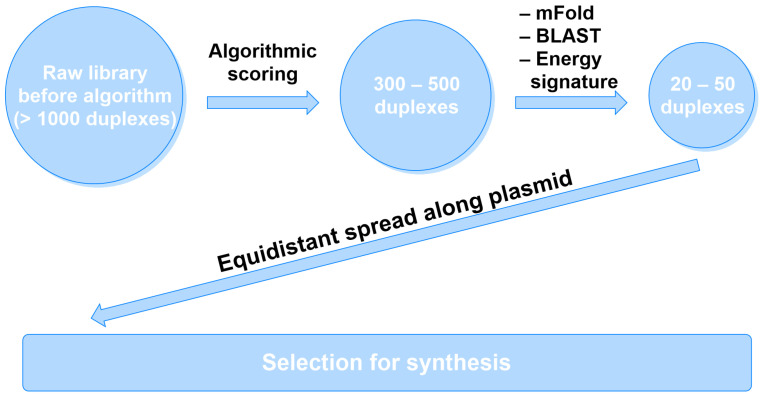
The principal scheme of the algorithm for the siRNA design. The raw library underwent algorithmic scoring by the list of predefined parameters (see Section 4.1). Then, the sequences were tested by NCBI-nBLAST (megablast) (https://blast.ncbi.nlm.nih.gov/Blast.cgi, accessed on 6 November 2025) and mFold (https://www.unafold.org/, accessed on 6 November 2025) to minimize possible off-target effects and exclude thermodynamically rigid variants to determine the final pool of siRNAs. Lastly, the novel “energy signature” algorithm for siRNA modification prediction was applied to each unmodified siRNA to suggest modification (see Section 4.2). The unmodified and modified siRNAs were synthesized and tested by in vitro studies.

The aim of this study is to develop and validate the approach to the design and modification of siRNAs against the mRNAs of target genes *ITGB1* and *CD47*, considering patterns of their thermodynamic stability, as well as performing a synthesis and modification of the most promising molecules and identifying the lead candidates during in vitro research. The principal scheme of the algorithm is depicted in Figure 1, and the work of the algorithm is described in detail in Section 4.

## 2. Results

### 2.1. Design of siRNA Sequences

The use of the described algorithm yielded libraries of siRNA sequences against the mRNAs of *ITGB1* and *CD47* genes, comprising 735 and 1055 duplexes, respectively. The sequences were scored, and siRNAs with scores less than 10 were filtered out, shrinking the libraries to 100 duplexes against each gene. Further use of the method decreased the size of the library to 20 specimens each.

The total libraries with scores are presented in the Supplementary Materials. Out of them, the six most promising duplexes were selected for each gene in such a way that they equally divide the span of the coding sequence. Selected sequences are presented in Table 1.

### 2.2. In Vitro Screening of Ribose Duplexes

**Figure 2:**
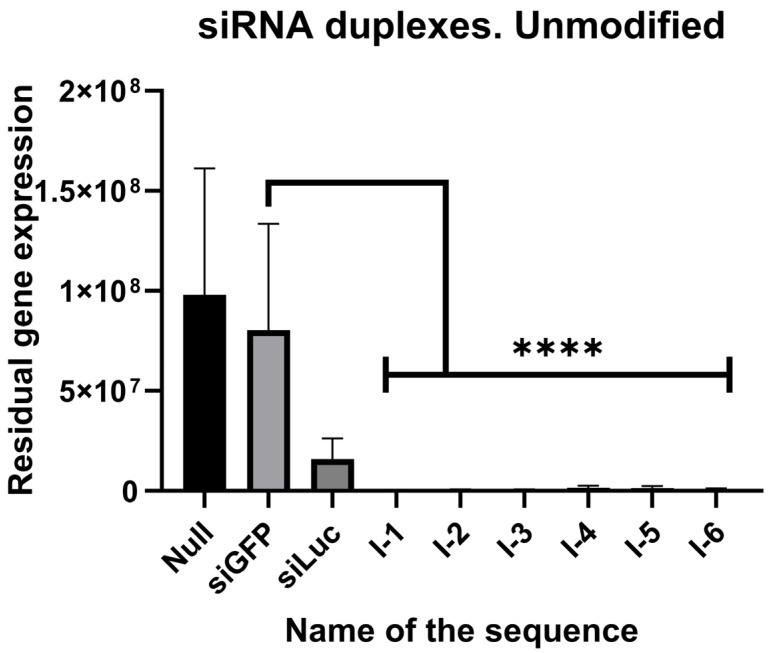
Efficacy of unmodified siRNAs against *ITGB1* in HEK293T cell line. Null—null control, siGFP—negative control, siLuc—positive control, I-1, I-2, I-3, I-4, I-5, I-6—studied siRNAs, N = 5, ****—*p*-value < 0.00001, significance determined by Kruskal–Wallis test.

Owing to the position of the target gene downstream of the luciferase reporter gene, it is reasonable to determine their expression by the level of luciferase luminescence. Ribose duplexes were subjected to an in vitro study according to Section 4. The efficacy of the molecules was determined. For the *ITGB1* gene, the results are depicted in Figure 2 and stated in Table 2. All of the siRNAs decreased the expression of the target gene at least 50-fold. The most effective siRNA appeared to be I-1, inhibiting the expression 250-fold.

**Figure 3:**
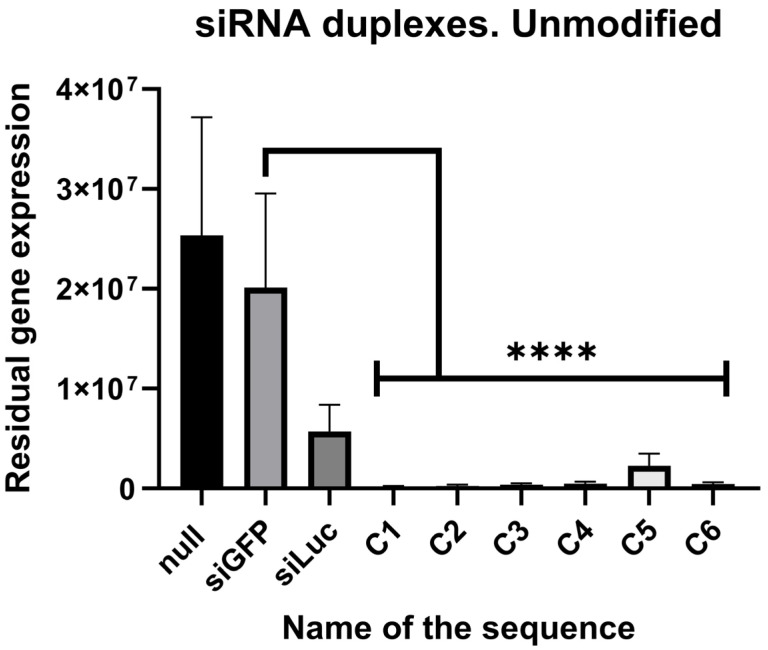
Efficacy of unmodified siRNAs against *CD47* in HEK293T cell line. Null—null control, siGFP—negative control, siLuc—positive control, C1, C2, C3, C4, C5, C6—studied siRNAs, N = 5, ****—*p*-value < 0.00001, significance determined by Kruskal–Wallis test.

For the *CD47* gene, the results are depicted in Figure 3 and stated in Table 3. All of the siRNAs decreased the expression of the target gene at least 10-fold. The most effective siRNA appeared to be C-1, inhibiting the expression 111-fold.

### 2.3. Design of siRNA Modifications

**Figure 4:**
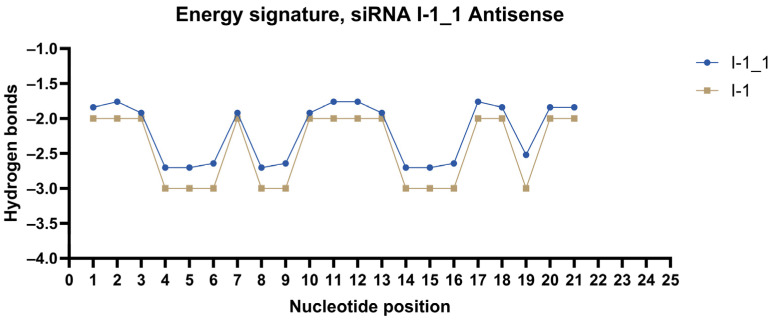
Energy signature of antisense strand of siRNA I-1_1.

**Figure 5:**
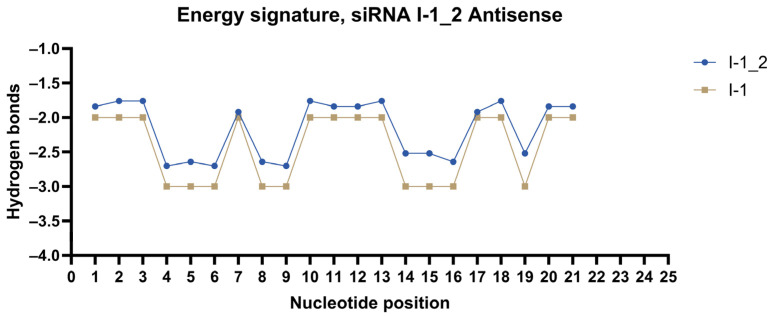
Energy signature of antisense strand of siRNA I-1_2.

**Figure 6:**
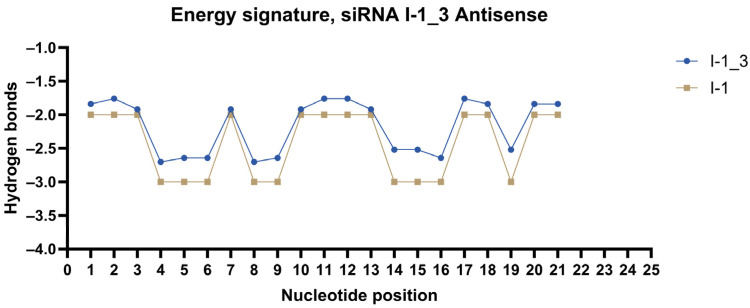
Energy signature of antisense strand of siRNA I-1_3.

Energy signatures were plotted for each strand of the most promising duplexes. siRNAs were modified according to the method. Modification patterns are presented in Table 4. Energy signatures for a subset of siRNAs against the mRNA of *ITGB1* are presented in Figure 4, Figure 5 and Figure 6 in comparison to the unmodified strand.

### 2.4. In Vitro Screening of Modified siRNAs

**Figure 7:**
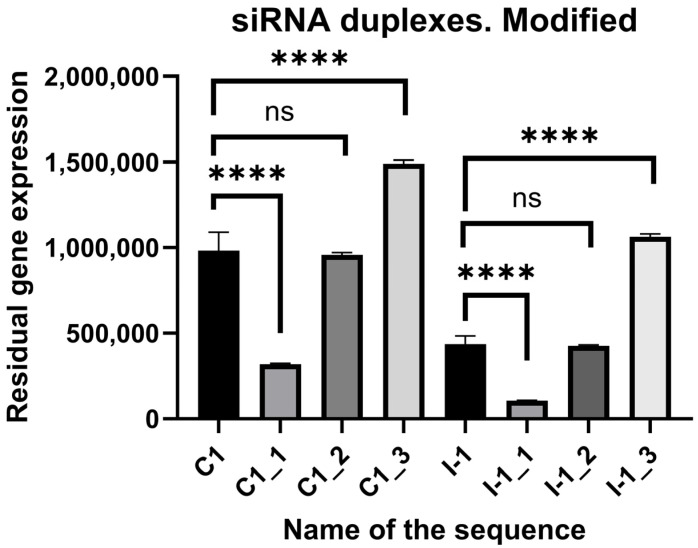
Efficacy of modified siRNAs against *CD47* and *ITGB1* in the HEK293T cell line compared to the unmodified originators C1, I-1—studied unmodified siRNAs, C1_1, C1_2, C1_3, I-1_1, I-1_2, I-1_3—studied modified siRNAs. N = 5, ns—statistically non-significant difference, ****—*p*-value < 0.00001, significance determined by Kruskal–Wallis test.

Subjecting modified siRNAs to in vitro research in the confines of the same model yielded the efficacy of the molecules. According to the results of the study, modifying the strands of the siRNA according to the first method increased the efficacy of the molecule at least three-fold. The second approach to modification did not significantly impact the efficacy of the siRNA, whereas the third approach to modification decreased the activity by up to 1.5 times. The results are depicted in Figure 7.

## 3. Discussion

Undoubtedly, widely available software provides significant opportunities in siRNA design. However, the provided output is a huge array of duplexes. It is almost inconceivable to be able to evaluate all the sequences in vitro, let alone in vivo. Therefore, in silico selection is of high importance, allowing for the streamlining of the whole research process. According to the results presented in Section 2.2, all of the siRNA possesses high efficacy, which is a drastically increased success rate compared to our previous research, in which only 2 out of 10 siRNAs were deemed efficient [18].

In the next step of the research, the best performing siRNAs were subjected to chemical modifications. In the framework of this study, we implemented 2′-OMe- and 2′-F-substituted deoxyribonucleotides due to their being readily commercially available. It must be noted that there is an overwhelming amount of chemically modified nucleotides. Yet, these two modifications remain the prime candidates due to their accessibility and effectiveness. Generally speaking, the implementation of the chemically modified nucleobases does not only limit the rate of the oligonucleotide degradation by environmental enzymes, but also improves their thermodynamic properties due to the decreased variability of the sugar–nucleoside bond. The substitution of 2′-ribose hydroxyl groups exhibiting different steric and/or electronic effects may hinder the mobility of the nucleoside–sugar bond, increasing the probability of the correct orientation of the nucleobase for the coupling with its complementary pair. Moreover, 2′-deoxyribose substituents may affect the keto-enolic tautomeric effects, which further influence the quality of the pairing. Therefore, formed hydrogen bonds exhibit increased stability and are formed more quickly.

Furthermore, previous modification of the siRNAs was directed by the empirical evidence being, frankly speaking, almost random, without significant consideration of the intricate molecular kinetics of the cellular processes. However, the received results after the modification of the siRNAs according to the methods described in Section 4.2 show curious dependencies. It appears that each of the methods used leads to different results; for example, the first suggested method improves the siRNA’s efficacy, whereas other methods do not significantly impact or even drastically decrease the efficacy. Therefore, it is reasonable to draw the conclusions that the number of positive gradients should be more than the number of negative gradients by 1–2, there should not be more than one plateau, and the 5′-end should incorporate low-energy islets followed by a negative gradient for a 3′-end, and vice versa. More details on the modifications process are available in Section 4.2.

Moreover, in our previous research, which will be published shortly, we noted that there is no strict correlation between the efficacy of unmodified siRNAs and the efficacy of optimally modified siRNAs. We had designed 10 siRNAs against the mRNA of the *PCSK9* gene. The results showed that the most efficient molecules were P1, P2, and P6, decreasing the expression of the target gene 2.5-fold, 2.8-fold, and 1.8-fold, respectively.

However, after applying the described method to these siRNAs, the duplex P6-7 significantly outperformed the previous lead candidate, exhibiting a 37-fold inhibition by P2-12 and a 100-fold inhibition by P6-7.

The received data encouraged the formation of the hypothesis that the energy signature of the strands significantly influences the behavior of the cellular machinery. It is supposed that the energy signature of the antisense strands directs the spatiotemporal kinetics of the RISC against the target mRNA.

Recently, profound studies were published considering the importance of the 10th and 11th nucleotides of the antisense strand in the process of RNAi [19], as well as an overall review of the thermodynamic behavior of the duplex [20]. Moreover, it is helpful to consider other RNA-dependent processes of matrix synthesis. It is widely acknowledged that, in the process of translation, the amino acid sequence is determined by the coupling of the codon on the mRNA and an anticodon on the tRNA. However, it is rather curious how the resulting amino acid is determined considering the energy signature of the anticodons. It appears that the anticodons are quite frequently modified by the introduction of 5′-Me-C and 2′-OMe substitutes, as well as thiolated phosphodiester bonds [21]. Analysis of amino acid families suggests that the main driving force for their coupling with the corresponding codons is not the monomers themselves but the arrangement of low-energy and high-energy monomers [22].

This data provides substantial evidence for the hypothesis, suggesting that the RISC is likewise independent of the concrete monomer but relies on the energy signature.

Admittedly, HEK293T is not a hepatocellular cell line. However, this cell line is supreme for the primary in vitro screening of siRNA activity due to it being specifically cultivated to withstand transfection procedures well. Nevertheless, since the ultimate aim of this research is the development of an siRNA-based drug to treat hepatocellular carcinoma, testing on hepatocellular carcinoma cell lines like Huh7, HepG2, Hep3B, and/or others is required. While *ITGB1* and *CD47* are implicated in hepatocellular carcinoma, therapeutic efficacy in HCC models was not evaluated here and will require future validation in hepatocyte-derived and in vivo systems. In the framework of this research, we have attempted to evaluate the effectiveness of our siRNA candidates in Huh7 (CVCL_0336); however, the results were not susceptible to interpretation due to the poor survivability of the cells under the action of the transfecting agent. Therefore, we decided to postpone this experiment until we receive the conjugate of our candidate siRNAs with carrier molecules (e.g., GalNAc), which would enable transfection without any external agents.

## 4. Materials and Methods

### 4.1. Design of siRNA Sequences

To date, there is a vast choice of software capable of siRNA design. Generally, they are based on foundational studies of Ui-Tei, Reynolds, and Amarzguioui [23,24,25]. We fused these approaches and validated our own algorithm for siRNA design, which was used in this work [26]. The application of the algorithm generated sequences of antisense and sense. The algorithm fuses named approaches and implements a basic scoring system. Each siRNA is assigned a score based on the following criteria (Table 5).

The application of the scoring system shrunk the sizes of the libraries to the 100 most promising duplexes for each gene. Then, the sequences of the strands were submitted to NCBI-nBLAST (megablast) to minimize possible off-target effects, with the lower threshold of homology being 80% [27]. Afterwards, siRNAs annealing to thermodynamically rigid segments of mRNA predicted by mFold [28] were excluded.

### 4.2. Modification of the siRNAs

After the in vitro study, lead ribose candidates were identified. In order to design patterns of chemical modifications for them, a novel approach was used. It relies on the visual representation of thermodynamic patterns of the strands we call the “energy signature”. Monomers were separated into “low-energy” monomers—A and U and “high-energy” monomers—G and C. For them, abstract energy values were assigned according to the amount of hydrogen bonds formed: low-energy monomers—2; high-energy monomers—3. In the framework of this research, only 2′-OMe and 2′-F—substituted monomers—were used. We reasoned that the addition of these modifications decreases the strength of hydrogen bonds; specifically, the addition of 2′-OMe decreases the non-covalent interactions 0.92-fold, 2′-F, 0.88-fold. For DNA monomers, the non-covalent interactions are stronger than 1.1-fold. 2′OMe-C is noted as an outlier, decreasing the strength of the interaction 0.84-fold compared to the ribose counterpart. For now, the energy signature model is a heuristic representation intended to guide relative thermodynamic asymmetry rather than a quantitative free-energy calculation.

Energy signatures of the stands were plotted in the IV quarter of the coordinate plane; along the abscissa axis, the positions of the monomers were plotted, along the ordinate axis, the energy of the interaction. As a result, a unique graph for every strand was formed.

**Figure 8:**
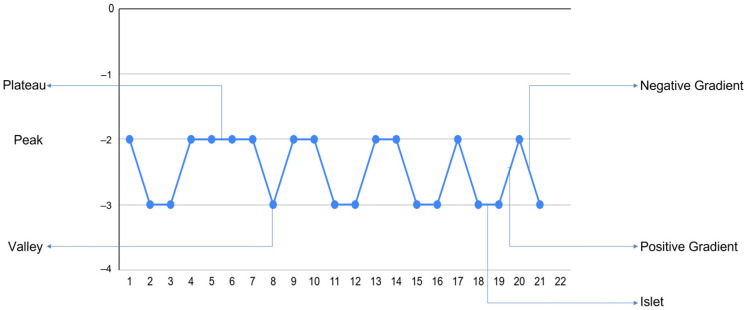
The structural elements of the energy signature.

The graph can be subdivided into structural elements. The transition between monomers of different energies was called the “gradient”: from low-energy to high-energy—negative; from high-energy to low-energy—positive. Gradients connected by a single low-energy monomer were called “peaks”; high-energy—“valleys”. Peaks or valleys comprising 2–3 monomers were called “islets”; 4 or more—“plateaus”. The structural elements are depicted in Figure 8.

Three distinct ways of altering the energy signatures were approached. The first way was focused on the introduction of positive gradients into islets and plateaus towards the 3′-end of the antisense strand and vice versa for the sense strand, as well as on increasing the values of the existing gradients. The second way was focused on creating subdivisions of islets and plateaus by the introduction of minute peaks or valleys. The third way was opposite to the first.

For all strands, the generation of a maximal gradient between the 10th and 11th nucleotides was attempted.

### 4.3. Automated Solid-Phase Oligonucleotide Synthesis, Chromatographic Purification, Physico-Chemical Characterization

Oligonucleotide strands were chemically synthesized in the process of solid-phase oligonucleotide synthesis on an automated DNA/RNA synthesizer PolyGen (PolyGen GmbH, Langen, Germany) according to the described methods [29]. Strands were purified during reverse-phase chromatography on GlenPak cartridges (Glen Research, Sterling, VA, USA) according to the instructions of the manufacturer [30]. The sequence of the synthesized molecules was confirmed by MALDI-ToF mass-spectrometry on Bruker MALDI microflex 400 (Bruker Optik GmbH, Ettlingen, Germany) according to the laboratory regimen.

Afterwards, corresponding strands were equimolarly mixed and annealed in a thermoshaker ICLEAR CHL-M3000 (Being Technology Co., Ltd., Kunshan City, Jiangsu Province, China) at T = 95 °C for 5 min and left to cool down to room temperature.

### 4.4. Reporter Plasmids

**Figure 9:**
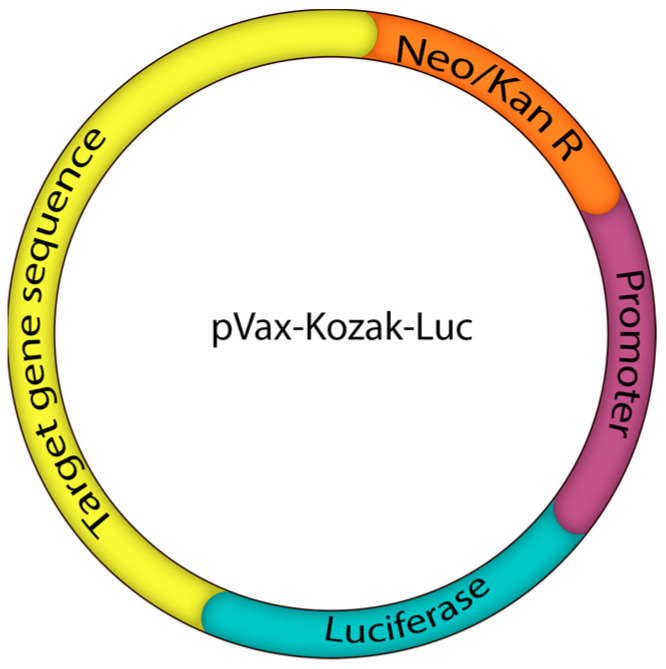
Scheme of reporter plasmids. Target genes—mRNA sequence of *ITGB1*, *CD47*, promoter—CMV promoter, Luciferase—luciferase reporter gene (*Luc*), Neo/Kan R—gene for neomycin/kanamycin resistance.

For the use in the in vitro studies, reporter plasmids were designed and created by Evrogen (Moscow, Russia). The plasmids comprised the mRNA sequence of the studied genes (*ITGB1* NM_133376.3; *CD47*–NM_198793.2) fused with the lucfierace (luc) reporter gene under a single CMV promoter. The plasmids were supplied with the gene for neomycin/kanamycin resistance to enable amplification (Figure 9).

### 4.5. In Vitro Study

To evaluate the efficacy of suppression of the expression of the target gene, a non-infectious model of gene expression was created. In the model, HEK 293T cells (CVCL_0063) were used. The cell line was kindly provided by the Institute for Molecular Biology of the Russian Academy of Sciences (IMB RAS). The cells were planted in the amount of 100 thousand cells per well into a 24-well plate (SPL, Naechon-Myeon, Pocheon-si, Gyeonggi-do, Republic of Korea) in full DMEM medium (PanEco, Moscow, Russia) with the addition of 10% veal embryonic serum, 300 mg/L L-glutamine (PanEco, Moscow, Russia), 25 mM HEPES (PanEco, Moscow, Russia), 50 µg/mL gentamycin (Gibco, Grand Island, NY, USA), and 10 mM sodium pyruvate (Servicebio, Wuhan, Hubei Province, China). The plate was incubated at 37 °C in an atmosphere of 5% CO_2_. Then, the cells were transfected with a mixture of 0.2 µg of plasmid and 0.4 µg of siRNA with 1 µL of Lipofectamine 3000 (Thermo Fischer Scientific, Waltham, MA, USA) within 100 µL of optiMEM medium (Gibco, Grand Island, NY, USA).

One of the wells was only transfected by the plasmid as a null control, one by non-specific siRNA (siGFP) as a negative control, and one by siRNA specific to the reporter sequence (siLuc) as a positive control. Each siRNA was tested in 5 biological repetitions. The plate was incubated at 37 °C in the atmosphere of 5% CO_2_ for 24 h. Afterwards, cells were lysed and the luciferase activity was evaluated by the Bright-Glo™ Luciferase Assay System (Promega, Madison, WI, USA) kit.

### 4.6. Statistical Evaluation

The results were statistically evaluated with GraphPad Prism (version 10.4.0 (621)) software (GraphPad Software, Boston, MA, USA) using embedded two-way ANOVA analysis by the non-parametric Kruskall–Wallis criterion.

## 5. Conclusions

Here is suggested an in silico approach to siRNA design and modifications. We demonstrated its efficacy applied to HCC-related markers in simple studies. By computational modeling, we have identified several potent siRNA candidates as well as their modifications for further complex in vivo research. The suggested approaches help to save a lot of time and resources for the prompt screening of potential siRNA candidates. We achieved a drastic improvement in the success rate of the identification of molecules capable of a more than 50-fold inhibition of the expression of the targeted gene. This study establishes a robust design and optimization strategy suitable for subsequent validation in hepatocellular carcinoma models.

The obtained results also prompted us to delve into the intricacies of the complex mechanisms of RNA interference as a structured process. Identifying specific relationships between the structure and properties of molecules helped us to make the most successful modifications to candidate molecules.

Of course, the proposed algorithm is only an early development and will require further, more in-depth research to determine its limitations. However, it can already be said with confidence that the development and structuring of the siRNA modeling process will make a significant contribution and speed up the process of searching for and testing siRNAs to meet various targets and challenges.

## Figures and Tables

**Table 1:** Selected siRNAs against the mRNAs of *ITGB1* (I-) and *CD47* (C).

Name	Antisense 5′-3′	Sense 5′-3′
I-1	UUUGCAUUCAGUGUUGUGGGA	CCACAACACUGAAUGCAAAGU
I-2	UUAAAAGCUUCCAUAUCAGCA	CUGAUAUGGAAGCUUUUAAUG
I-3	UCCUUAACUAGCUAGCAGGAC	CCUGCUAGCUAGUUAAGGAUU
I-4	UAAAAAGGCAAAUUGACCGCU	CGGUCAAUUUGCCUUUUUAAU
I-5	AAAACAAGAAUGUGACUAGUG	CUAGUCACAUUCUUGUUUUAA
I-6	AAUACAUCAGAGUCAAGACAU	GUCUUGACUCUGAUGUAUUUU
C1	UAACUAACAAUCACGUAAGGG	CUUACGUGAUUGUUAGUUAAG
C2	UGACAGUGAUCACUAGUCCAG	GGACUAGUGAUCACUGUCAUU
C3	UUUUCAUAGAUAUCUCUGGGU	CCAGAGAUAUCUAUGAAAACC
C4	UCAAGAAGAGCUGUCUUGCUA	GCAAGACAGCUCUUCUUGAAA
C5	UUAAAAGGUACAACUUUAGUU	CUAAAGUUGUACCUUUUAAUA
C6	AGUGCAAAUAACAAUUUGGUG	CCAAAUUGUUAUUUGCACUAA

**Table 2:** Fraction of residual gene expression after treatment with unmodified siRNAs against *ITGB1*.

pVax + 0	siGFP	siLuc	I-1	I-2	I-3	I-4	I-5	I-6
1	0.816	0.183	0.004	0.005	0.005	0.018	0.016	0.008

**Table 3:** Fraction of residual gene expression after treatment with unmodified siRNAs against *CD47*.

Null	siGFP	siLuc	C-1	C-2	C-3	C-4	C-5	C-6
1	0.796	0.235	0.009	0.015	0.018	0.0273	0.098	0.022

**Table 4:** Modification pattern of siRNAs.

Name	Antisense 5′-3′	Sense 5′-3′
I-1	rUrUrUrGrCrArUrUrCrArGrUrGrUrUrGrUrGrGrGrA	rCrCrArCrArArCrArCrUrGrArArUrGrCrArArArGrU
I-1_1	mUmUmUmGmCmAmUmUmCmAmGmUmGmUmUmGmUmGmGmGmA	mCmCmAmCmAmAmCmAmCmUmGmAmAmUmGmCmAmAmAmGmU
I-1_2	mUfUfUmGmCmAfUmUmCfAmGmUmGmUfUmGmUmGfGfGmA	fCfCmAmCmAmAmfCmAmCmUfGmAmAmUmGfCmAmAmAmGmU
I-1_3	fUfUmUmGmCmAfUfUmCfAmGmUmGmUfUmGmUmGfGfGmA	fCdCmAmCmAmAfCmAmCdTmGmAmAmUmGfCfAmAmAmGmU
C1	rUrArArCrUrArArCrArArUrCrArCrGrUrArArGrGrG	rCrUrUrArCrGrUrGrArUrUrGrUrUrArGrUrUrArArG
C1_1	mUmAmAmCmUmAmAmCmAmAmUmCmAmCmGmUmAmAmGmGmG	mCmUmUmAmCmGmUmGmAmUmUmGmUmUmAmGmUmUmAmAmG
C1_2	mUfAmAmCmUfAmAmCmAfAmUmCmAfCfGmUfAmAmGfGfG	fCfUfUmAmCmGmUmGfAfUmUmGmUmUmAmGmUmUmAfAfG
C1_3	mUmAfAmCmUmAfAmCmAfAmUmCmAfCfGmUmAfAmGmGdA	fCdTmUmAmCmGmUmGmAdTmUmGmUdTmAmGmUmUmAfAfG

m—2′-OMe, f—2′-F, d—deoxyribose, r—ribose.

**Table 5:** Algorithmic scoring system.

Criterion	Value	Score
Content of GC in the antisense strand	36–52% of the strand	1
Amount of GC and AU repeats in the antisense strand	GC repeats < 3, AU repeats < 4	1
Low GC content in 9–14 positions of antisense strand	GC content in 9–14 < GC_content_/2	2
Number of A/U in positions 13–19 of the antisense strand	At least 3	1
G/C at position 1 of the sense strand	Yes	1
A/U at position 10 of the sense strand	Yes	1
A at positions 3 and 19 of the sense strand	Yes	1
Absence of G/C at position 19 of the sense strand	Yes	1
Absence of G at position 13 of the sense strand	Yes	1
A/U at position 1 of the antisense strand	Yes	1
A at position 6 of the antisense strand	Yes	1
Content of GC in positions 2–7 and 8–18 of the antisense strand	GC_content_ in positions 2–7 < 19%, GC_content_ in positions 8–18 < 52%	1
Successful mFold	No annealing to thermodynamically rigid segments of mRNA	1
Successful BLAST	No significant off-target effects	2

G–guanine, C–cytosine, A–adenine, U–uracil, GC_content_—content of GC in the antisense strand.

## Data Availability

The original contributions presented in this study are included in the article/Supplementary Materials. Further inquiries can be directed to the corresponding author.

## Supplementary Materials

The following supporting information can be downloaded at https://www.mdpi.com/article/10.3390/ijms27020603/s1.

## Abbreviations

The following abbreviations are used in this manuscript:

ANOVA	Analysis of Variance
ASO	Antisense Oligonucleotide
CD	Cluster of Differentiation
CMV	Cytomegalovirus
CTLA	Cytotoxic T-Lymphocyte-Associated Protein
DNA	Deoxyribonucleic Acid
DMEM	Dulbecco’s Modified Eagle Medium
ECAM	Extracellular Adhesion Molecule
FDA	Food and Drug Administration (USA)
FMBA	Federal Medical–Biological Agency (Russia)
HCC	Hepatocellular Carcinoma
HEK	Human Embryonic Kidney
HEPES	(4-(2-hydroxyethyl)-1-piperazineethanesulfonic acid)
IAP	Integrin-Associated Protein
ITGB	Integrin Subunit Beta
mRNA	Messenger RNA
MALDI-ToF	Matrix-Assisted Laser Desorption/Ionization—Time of Flight Mass Spectrometry
miRNA	MicroRNA
NCBI	National Center for Biotechnology Information
NRC	National Research Center
PD-1	Programmed Cell Death Protein 1
PD-L1	Programmed Death-Ligand 1
RNA	Ribonucleic Acid
RNAi	RNA Interference
RISC	RNA-Induced Silencing Complex
SIRPα	Signal Regulatory Protein Alpha
siRNA	Small Interfering RNA
UTR	Untranslated Region
2′-F	2′-Fluorinated nucleotide
2′-OMe	2′-O-Methylated nucleotide
